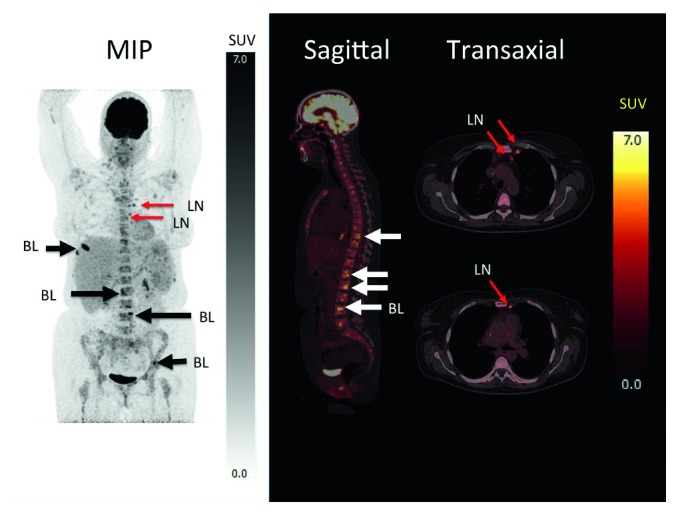# Classical Hodgkin Lymphoma Presenting with Severe, Recurrent Hypothermic Episodes

**DOI:** 10.1155/2018/3726593

**Published:** 2018-09-25

**Authors:** Jonas Juul Hansen, Hans Beier Ommen, Lars Christian Gormsen, Francesco Annibale d'Amore, Peter Martin Hjørnet Kamper

**Affiliations:** ^1^Department of Hematology, Aarhus University Hospital, Tage-Hansens Gade 2, DK-8000 Aarhus, Denmark; ^2^Department of Nuclear Medicine and PET Center, Aarhus University Hospital, Nørrebrogade 44, DK-8000 Aarhus, Denmark

## Abstract

We report a case presenting with recurrent episodes of severe hypothermia preceding the diagnosis of Hodgkin lymphoma. The episodes of hypothermia were accompanied by other symptoms of autonomic dysfunction, mainly hypotension, which could be caused by autonomic neuropathy as part of a paraneoplastic syndrome. In comparison with previous reports describing an association between the presence of hypothermia and an adverse outcome, the present patient has responded well to lymphoma-specific treatment and is currently in an ongoing complete remission. Due to the peculiar cyclic pattern of the hypothermic episodes presented in this case, we hypothesize whether intermittent release of disease-related chemo- and cytokines could be a plausible pathogenetic explanation.

## 1. Introduction

Hypothermia in relation to Hodgkin lymphoma (HL) is a rare phenomenon, and to the best of our knowledge, this is among the only 18 cases reported worldwide, where a cyclic pattern of hypothermia is described [1]. In the previously reported cases of HL-associated hypothermia, the hypothermia had mostly occurred after chemotherapy and the administration of various drugs, for example paracetamol or prednisone [2–10]. Only in a small subset of these cases, hypothermia was reported to develop prior to drug administration. Hence, episodes of hypothermia not preceded by the administration of paracetamol, prednisone, or chemotherapy are rare.

## 2. Case Presentation

A 39-year-old woman was admitted to the ER with a right-sided drop-foot, fever, and pancytopenia. The patient reported night sweats during the last month and a 25 kg weight loss over the last year. On physical examination, enlarged, painless right cervical and axillary lymph nodes were found; the largest of which was 1 × 2 cm. A CT-scan revealed marginally enlarged axillary and abdominal lymph nodes as well as a marginally enlarged spleen. A bone marrow biopsy was performed and bilineage dysplasia was found, possibly suggestive of a myelodysplastic syndrome. The fever and anaemia responded well to broad-spectrum antibiotics and blood transfusions, respectively. An EBV-viremia (18.500 DNA copies/mL) was detected, and the patient was treated with acyclovir followed by rituximab. The decision to initiate acyclovir and rituximab was taken due to suspected virus-associated haemophagocytic syndrome. During this treatment, three separate episodes of hypothermia occurred with an interval of approximately two weeks between each episode.

**Figure 1:**
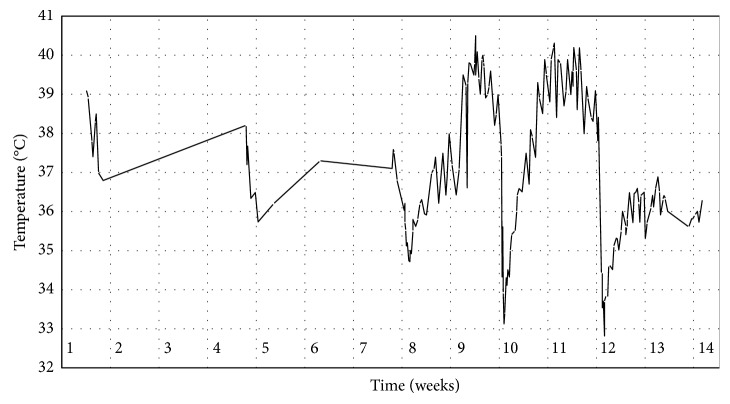


The first episode occurred the day after rituximab infusion and was accompanied by moderate bradycardia, hypotension, and a prolonged QT interval. The patient was subjectively unaffected. However, the patient had a syncope-like episode a few days later. Subsequent ECG monitoring at the Department of Cardiology did not reveal any arrhythmias. The second episode of hypothermia occurred 13 days later just before a planned rituximab infusion. However, the patient had already received paracetamol as premedication prior to rituximab. The patient only experienced mild symptoms related to hypothermia, that is, moderate sweating, moderate hypotension, and insecure gait. Intravenous fluids were administered with clinical effect. During the third episode, an ear temperature as low as 32.8°C was recorded. The patient experienced profuse sweating and an ECG demonstrated bradycardia along with a borderline prolonged QT interval. The blood pressure reached a low point of 85/52 mmHg and the pulse was recorded as 50 bpm. Similar to the previous episodes, the patient was relatively unaffected, and no specific treatment in order to increase the body temperature was initiated. The third episode differed from the first two since neither rituximab nor paracetamol was given prior to the onset of hypothermia (Figure 1).

A PET/CT was performed, revealing increased FDG uptake in enlarged right cervical and mediastinal lymph nodes and in focal areas of the spleen and liver. Furthermore, increased FDG uptake was found at multiple skeletal sites and in the bone marrow, but without CT correlate (Figure 2). A surgical biopsy from a right cervical lymph node revealed classical HL, nodular sclerosis type. The bone marrow biopsy was without morphological and immunophenotypic evidence of lymphoma.

In the weeks following the last episode of hypothermia, three separate attacks with generalized seizures occurred. One of them was clinically described as a classic tonic-clonic Jackson-type attack, while the other two were less well characterized. An EEG showed focal activity compatible with epileptic state. Cerebral CT and MRI scans did not show any sign of intracranial or intraspinal tumors and did not provide any clues to clarify why the attacks occurred.

The patient was treated with 8 series of BEACOPP-14. The peroneal palsy which had remitted worsened again during chemotherapy, and it was therefore decided to continue without vinca alkaloids from series 3 and onwards. After 8 series the PET scan revealed a complete metabolic remission. No radiotherapy was added. Although no repeat bone marrow biopsy was done upon completion of the treatment schedule, all blood counts returned to normal making the initial diagnosis of a possible myelodysplastic syndrome unlikely. The patient received her last round of chemotherapy on December 2015. She was still in 1st complete remission and is regularly followed on an outpatient basis. No additional episodes of hypothermia have been registered so far.

## 3. Discussion

The biological mechanisms leading to the development of hypothermia in association with HL are poorly understood. A direct structural involvement of the hypothalamus could potentially affect the temperature regulation, but no imaging evidence of such an involvement was found in our case or any of those previously described [1]. Autonomic neuropathy has also been proposed as a possible mechanism, and this theory is supported by the fact that the previously described hypothermic episodes often have occurred along with other symptoms of autonomic dysfunction [1]. Furthermore, a review concluded that the spectrum of clinical manifestations of paraneoplastic neuropathy is broad and may well include a variety of autonomic dysfunctions [11]. No systematic evaluation of the autonomic functions was performed in the present case, but the presence of concurrent hypotension, bradycardia, and syncope makes this a plausible explanation. Neurotoxicity is a known adverse effect of certain chemotherapeutical substances, for example, vincristine and vinblastine, and in the majority of the previously reported cases, the chemotherapy was administered prior to the episodes of hypothermia [10]. The vinca alkaloids are most likely to induce peripheral neuropathy but have also been implicated as a risk factor for the development of autonomic dysfunction [12]. In the present report, the episodes of hypothermia developed prior to the administration of chemotherapy, demonstrating that, in our patient, other mechanisms must be involved in the development of the hypothermic episodes. Interestingly, in an observation study by Bilora et al. it was concluded that a paraneoplastic phenomenon was the most likely explanation of the autonomic dysfunction observed in Hodgkin lymphoma [13]. At the time of the episodes of hypothermia, no diagnosis of malignant disease had yet been made. Therefore, the possibility of hypothermia being a paraneoplastic phenomenon was not considered at the time and consequently no serologic tests or nerve biopsy was made.

Hypersensitivity to antipyretics has also been suggested as a plausible mechanism [1]. In our case, paracetamol was administered prior to at least one of the episodes of hypothermia. However, three distinct episodes of hypothermia were observed in the present case and at least one of the episodes was not preceded by administration of neither chemotherapy nor paracetamol.

EBV has previously been suggested as a possible cause of neurological symptoms such as autonomic dysfunction and peripheral neuropathy [14]. In the present case, the episodes of hypothermia demonstrated a recurrent pattern, which did not resemble the temperature pattern usually observed in an EBV infection. Whether EBV may have a role in the pathogenesis of the reported symptoms in general, or hypothermia in particular, is unclear. However, in this case the reported cyclic pattern of hypothermia seems more similar to the characteristic pattern observed in HL-associated Pel-Ebstein fever, and thereby more likely to be a lymphoma-associated paraneoplastic phenomenon [15].

It is relevant to notice that our patient has responded well to lymphoma-specific treatment and is in an ongoing complete remission. This is in contrast to the cases previously reported, where the presence of hypothermia has been associated with an adverse outcome [1].

## Figures and Tables

**Figure 2:**